# Changes in elderly women's health-related quality of life following discontinuation of hormone replacement therapy

**DOI:** 10.1186/1472-6874-5-7

**Published:** 2005-05-16

**Authors:** Debra A Heller, Carol H Gold, Frank M Ahern, Kristine E Pringle, Theresa V Brown, Margaret R Glessner

**Affiliations:** 1 First Health Services/The PACE Program, 4000 Crums Mill Road, Suite 301, Harrisburg, PA 17112 USA; 2 Department of Biobehavioral Health, The Pennsylvania State University, 315 East Health and Human Development, University Park, PA 16802 USA; 3 Pennsylvania Department of Aging/The PACE Program, 555 Walnut Street, 5th Floor, Harrisburg, PA 17101 USA

## Abstract

**Background:**

Many women have discontinued hormone replacement therapy (HRT) in view of recent findings. The goal of this study was to determine if HRT discontinuation is associated with changes in health-related quality of life (HRQOL) in elderly women.

**Methods:**

We studied women enrolled in Pennsylvania's Pharmaceutical Assistance Contract for the Elderly (PACE) program, linking prescription claims with data from a longitudinal mail survey. HRQOL measures included the number of days out of the last 30 that physical health was not good and analogous measures for mental health, pain, and interference with activities, as well as a composite "healthy days" measure developed by CDC. Longitudinal analyses focused on 2,357 women who completed surveys in both 2002 and 2003, and who used HRT at baseline (mean age = 75.5, range = 65–102). Propensity scores were used to match HRT continuers and discontinuers according to HRT type, demographics, and baseline HRQOL. Analysis of covariance was used to compare HRQOL change in continuers and discontinuers.

**Results:**

Between 2002 and 2003, 43% of HRT users discontinued therapy. Analysis of covariance to examine HRQOL change revealed complex interactions with age. Discontinuers aged 65–74 reported greater increases in days in which mental health was not good (p < .05), fewer "healthy days" (p < .05), more days in which health interfered with activities (p < .01), and more days with pain (p < .01). Among women aged 75–84, HRT discontinuers reported more days in which physical health was not good (p < .01); no other significant effects were observed in this group. Relative to HRT continuers, discontinuers aged 85 and older experienced apparent HRQOL improvements following cessation, with fewer days in which physical health was not good (p < .01), fewer days of poor mental health (p < .05), and more "healthy days" (p < .01).

**Conclusions:**

These results suggest that there are substantial age differences in response to HRT discontinuation. While women aged 65–74 experienced apparent declines in HRQOL following HRT cessation, women aged 85 and older experienced relative improvements. The HRQOL declines observed among younger women underscore the importance of communication between clinicians and patients throughout the discontinuation process. These results also demonstrate the value of HRQOL surveillance as a component of health program administration.

## Background

Few health topics are currently associated with as much confusion as the issue of hormone replacement therapy (HRT) for postmenopausal women. Historically, the results of observational studies spanning several decades have suggested that HRT confers important cardiovascular benefits for postmenopausal women [1,2]. In July of 2002, however, the combination HRT component of the Women's Health Initiative (WHI) study-a randomized placebo-controlled trial-was halted due to an excess incidence of coronary events, stroke, pulmonary embolism, and breast cancer among women receiving estrogen and progestin in combination [3,4]. In February of 2004, the estrogen-only arm of the WHI study was similarly halted due to observed increases in the risk of stroke among women with prior hysterectomy who received unopposed estrogen [5].

The well-publicized halting of the WHI combination therapy trial, along with emerging evidence from other studies, sparked intense debate and ultimately led to an important paradigm shift with respect to HRT. Guidelines such as those released in 2002 by the U.S. Preventive Services Task Force stress that the harmful effects of HRT related to breast cancer and cardiovascular risk outweigh potential benefits such as increased bone density, and that HRT should therefore not be used for the prevention of chronic conditions in postmenopausal women [6]. The newer guidelines and warnings are in sharp contrast to HRT's cultural history and early marketing, which portrayed hormone replacement as fundamental for the preservation of health, youth, and femininity. Many older women who are now long past menopause initiated HRT decades ago with the belief that they would continue with the therapy for life. The recent shifts in guidelines and practice have therefore presented a dilemma for clinicians and patients, and many women have opted to discontinue HRT [7].

Enhanced quality of life is a frequently-cited benefit of HRT [8], but recent studies addressing quality of life have produced mixed results. A number of observational studies comparing HRT users and non-users have observed better psychological health and health-related quality of life among HRT users [9-11]. Recent randomized clinical trials, however, have reported fewer apparent benefits for postmenopausal women. For example, comparing HRT users and non-users in the Heart and Estrogen/Progestin Replacement Study (HERS), Hlatky and his colleagues found no evidence of a general benefit in quality of life associated with HRT [12]. Among women with menopausal symptoms such as flushing, however, HRT use produced improvements in mental health and depressive symptoms. Similar findings were reported by Hays et al. using WHI data [13]. Both the WHI and HERS studies concluded that, aside from women experiencing acute menopausal symptoms, HRT users did not differ from non-users with respect to quality of life. No published studies to date, however, have specifically examined the impact of HRT *discontinuation *on quality of life. In addition, relatively few HRT studies have focused explicitly on elderly women, but have instead focused on younger postmenopausal women, who are most likely to experience acute menopausal symptoms. Elderly women comprise an important segment of the population of HRT users, and more research on elderly HRT users is needed. It is important for clinicians to understand the potential changes in quality of life that may be associated HRT discontinuation, and how women of different ages may respond to HRT discontinuation.

The goal of this study was to determine if HRT discontinuation is associated with changes in health-related quality of life in a population of elderly women. We studied women enrolled in a state pharmaceutical assistance program for the elderly, linking prescription claims with data from a longitudinal mail survey that addressed health-related quality of life. The availability of longitudinal survey data for HRT continuers and discontinuers provides an important opportunity to examine the impact of HRT discontinuation on the quality of life of elderly women.

## Methods

### Study population

Subjects included women who were enrolled during 2002 and 2003 in Pennsylvania's Pharmaceutical Assistance Contract for the Elderly (PACE), a state program providing prescription drug assistance to elderly with low to moderate incomes. All PACE applicants complete a detailed application form prior to enrollment. Depending on their income, enrollees are then required to re-enroll either annually or biannually. For purposes of research and evaluation, PACE includes an optional two-page survey – the Survey on Health and Well-Being – with all new and renewal enrollment applications. The survey was approved by the Institutional Review Board of the Pennsylvania State University. Historical data suggest that most PACE cardholders view the survey positively, with annual response rates exceeding 70% [14].

In order to examine longitudinal associations, the present study focuses on women who completed both the 2002 and 2003 Surveys on Health and Well-Being, and who were HRT users at baseline (i.e., the time that they completed the 2002 survey) based on prescription claims review.

### Measurement of health-related quality of life

Health related quality of life (HRQOL), although variously defined, refers generally to those aspects of quality of life that are clearly related to either physical or mental health [15,16]. The U.S. Centers for Disease Control and Prevention (CDC) has described HRQOL as "an individual's or group's perceived physical and mental health over time" [15]. Over the last three decades, HRQOL has been increasingly recognized as an important component of health status that should be considered when evaluating the effectiveness of health programs and medical treatments [17].

The PACE Survey on Health and Well-Being includes a series of HRQOL questions that were adapted from the CDC's Behavioral Risk Factor Surveillance System (BRFSS) telephone survey. The BRFSS HRQOL measures include a core set of four questions as well as an optional set of questions related to pain perception and activity limitation [15]. Moderate to excellent retest reliabilities for the HRQOL module questions have been reported [18]. Although brief, the BRFSS HRQOL module has produced results comparable to the SF-36 in prior studies, and has successfully distinguished groups varying in clinical diagnoses [19,20]. Recent research has also demonstrated that the instrument is responsive to changes in health status and is well-accepted by older adults [21,22].

Table 1 displays the BRFSS HRQOL questions included in the PACE survey. The present analysis focuses on the number of days out of the last 30 that physical health was not good and analogous measures for mental health, interference of health with activities, and pain. The present study also utilized composite measures of "unhealthy days" and "healthy days" developed by CDC. The total number of unhealthy days in the last month was estimated by summing the number of "not good" physical and mental days, with a logical maximum of 30 days. CDC's measure of healthy days, a positive complementary form of unhealthy days, was computed by subtracting the number of unhealthy days from 30 [15,23]. Prior studies have demonstrated that the healthy and unhealthy days composite variables are valid and responsive measures of perceived health [15,19].

**Table 1:** Health-related quality of life questions included in survey

**Core HRQOL module questions**	
1.	Would you say that in general your health is: excellent, very good, good, fair or poor?
2.	Now thinking about your physical health, which includes physical illness and injury, for how many days during the past 30 days was your physical health not good?
3.	Now thinking about your mental health, which includes stress, depression, and problems with emotions, for how many days during the past 30 days was your mental health not good?
4.	During the past 30 days, for about how many days did poor physical or mental health keep you from doing your usual activities, such as self-care, work, or recreation?
**Optional HRQOL module questions (pain and activity limitation)**	

1.	During the past 30 days, for about how many days did PAIN make it hard for you to do your usual activities, such as self-care, work, or recreation?
2.	Are you limited in any activities because of any impairment or health problem?
3.	What is the MAJOR impairment or health problem that limits your activities?
4.	For HOW LONG have your activities been limited because of your impairment or health problem? Please give the length of time.
5.	Because of any impairment or health problem, do you need the help of other persons with your PERSONAL CARE needs, such as eating, bathing, dressing, or getting around the house?
6.	Because of any impairment or health problem, do you need the help of other persons in handling your ROUTINE needs, such as everyday household chores, doing necessary business, shopping, or getting around for other purposes?

### Measurement of HRT and other prescription drug use

PACE utilizes a point-of-sale claim payment system; at the time of dispensing, pharmacies submit electronic claims to PACE for adjudication and reimbursement. Complete data on all prescription medications obtained by study participants were therefore available for study, provided that participants filled the prescriptions by using their PACE cards. All HRT prescriptions, and details regarding the specific type of HRT, were identified by linking claim records for study participants to a database of drug attributes (Red Book^®^) provided by Medical Economics/Thomson Healthcare (Montvale, New Jersey). Systemic forms of HRT, including oral and transdermal products, were distinguished from topical forms on the basis of each dispensed product's formulation code.

HRT status was evaluated by identifying all systemic HRT claims dispensed between October 1, 2001 and March 31, 2004. PACE allows only a 30-day supply of medication to be dispensed per prescription refill. In order to allow for early refills and potential prescription overlap, baseline HRT users were defined as individuals who filled one or more HRT prescriptions during the 45 days preceding their 2002 baseline survey response date. For follow-up status evaluation, claims were examined for the entire time interval between the baseline survey date and up to 90 days after the 2003 follow-up survey date. Discontinuers were defined as individuals whose last HRT prescription was filled more than 45 days prior to their follow-up survey response date. Women who filled one or more HRT prescriptions during the 45 days immediately preceding the follow-up survey date were identified as continuers. A small number of baseline HRT users (n = 143) who did not have any HRT claims during the 45 days prior to the follow-up survey, but who filled HRT prescriptions within 90 days after their 2003 survey date, were also categorized as continuers. Baseline and follow-up HRT use for each study participant was further categorized as either unopposed estrogen (estrogen without progestin) or combination therapy (estrogen with progestin).

### Statistical analyses

#### Descriptive statistics

All statistical analyses were conducted using the Statistical Analysis System (SAS) for Windows, Version 8.2 (SAS Institute, Cary, NC). Descriptive statistics were used to describe HRT continuers and discontinuers in terms of demographic characteristics, self-reported health behaviors, HRT and other drug utilization, and HRQOL survey measures.

#### Propensity scores

The goal of the present study was to compare HRQOL change in HRT continuers and discontinuers. A limitation, however, is that women who choose to continue HRT may differ fundamentally from women who discontinue HRT. If so, then these fundamental differences may also be reflected in differential change in HRQOL, leading to biased estimates of the effect of HRT discontinuation on HRQOL. Such bias is inherent in many observational studies, in contrast to randomized trials in which subjects are assigned randomly to treatment or non-treatment groups.

Propensity scores have been proposed as a methodological strategy for bias control in observational studies [24-26]. The propensity score is defined as the conditional probability that an individual is a member of a treatment group, given all available covariate values [26]. Compared to traditional stratification, matching, or covariate adjustment methods, propensity scores offer the advantage of reducing a large number of background covariates to a single scalar value. Once propensity scores have been created, traditional procedures such as stratification or matching can then be applied to the propensity scores [26].

In the context of the present study, the propensity score represents the conditional probability (ranging from 0 to 1) that a woman using HRT at baseline will discontinue HRT, given her baseline characteristics. Propensity scores were created through a multivariate logistic regression analysis to predict the binary outcome of HRT discontinuation at the time of the follow-up (2003) survey, based on multiple explanatory variables measured at baseline (2002). The baseline explanatory variables included demographic measures (age, race, income, marital status, education, long-term care residence, and urban/rural residence), self-reported health behaviors (alcohol use and smoking), type of HRT, and non-drug utilization.

#### Matching of continuers and discontinuers

Continuers and discontinuers were matched using a SAS macro provided by researchers at the Mayo Clinic Division of Biostatistics [27]. The propensity score generated in the multivariate logistic regression described above was used as the primary matching factor. To control further for potential baseline differences in HRQOL, the baseline HRQOL measures were also subjected to principal components analysis, a data reduction technique that summarizes the variance shared by a set of variables [28,29]. The first principal component, which accounted for 71.9% of the total variance in the baseline HRQOL measures, was then used as a secondary matching variable. The rationale for including the principal component score in the matching algorithm was to ensure that the matched samples of continuers and discontinuers were well-balanced in terms of all baseline HRQOL measures. In addition to the propensity and baseline HRQOL principal component scores, continuers and discontinuers were further matched by baseline HRT type (estrogen alone or combination) and age group (65–74 years, 75–84 years, and 85 or older). Using the above-described selection factors, a single best-matching HRT continuer was identified for each HRT discontinuer. All subsequent analyses were conducted using the reduced sample of matched HRT continuers and discontinuers.

#### Analysis of covariance (ANCOVA)

Once the final matched sample of continuers and discontinuers was created, analysis of covariance (ANCOVA) was used to examine the impact of HRT continuation or discontinuation on HRQOL change. As discussed by Vickers and Altman [30], ANCOVA may be used to compare change in one or more groups by predicting follow-up scores from baseline scores and a treatment group indicator. In the present study, the ANCOVA model equation may be visualized as:

*HRQOL_2_*= *Constant *+ *β_1_·HRQOL_1_*+ *β_2_·HRT Group*

where HRQOL_1 _and HRQOL_2 _are baseline and follow-up HRQOL scores, β_1 _and β_2 _are coefficients to be estimated, and HRT Group is a binary variable coded 1 for HRT discontinuation and 0 for HRT continuation. Using the SAS GLM procedure, separate ANCOVA analyses were performed for each of the HRQOL questions. To explore age differences in response to HRT discontinuation, separate analyses were conducted within three age groups: 65–74 years, 75–84 years, and 85 years or older.

## Results

A total of 4,236 women completed a survey in 2002 and used HRT at baseline, based on one or more claims for systemic HRT products during the 45 days preceding their 2002 survey return date. Of these women, 1,076 (25.4%) had incomes low enough to qualify them for two years of PACE coverage rather than only one year, and were therefore not required to reapply for coverage in 2003. Among the remaining 3,160 women, 2,899 reapplied for PACE coverage in 2003, and 2,357 of those reapplying also completed the follow-up survey in 2003. The mean interval between the baseline and follow-up surveys was 367 days. Of the 2,357 baseline HRT users who completed surveys in both 2002 and 2003, 1,015 women (43.1%) discontinued HRT between the time of their baseline and follow-up surveys.

### Determinants of HRT discontinuation

Table 2 summarizes the results of a multivariate logistic regression analysis to predict HRT discontinuation from multiple baseline measures, including demographic factors, self-reported health behaviors, type of HRT, non-HRT prescription drug use, and baseline HRQOL. Percentage frequencies for each categorical measure and means for each continuous measure are shown for HRT continuers and discontinuers. Table 2 also presents the parameter estimates, standard errors, adjusted odds ratios with associated confidence limits, and probability values for all independent variables. The strongest predictor of HRT discontinuation was type of HRT used at baseline, with combination users being three times as likely as unopposed estrogen users to discontinue HRT (O.R. = 3.03, p < .0001). This result is not surprising given the fact that initial media attention focused on the WHI combination therapy arm that was halted in 2002; the unopposed estrogen arm of the WHI study was not halted until 2004. On average, HRT discontinuers had higher annual incomes than HRT continuers ($13,999 vs. $13,805, O.R. per $1,000 = 1.06, p = .0283). Relative to women living in urban areas, women residing in rural or semi-rural areas appeared more likely to discontinue HRT, although this result did not achieve conventional significance (O.R. = 1.17, p = .0948). Women who had any baseline use of cardiovascular drugs were significantly more likely than non-users to discontinue HRT (O.R. = 1.25, p = .0471), as were women who had any baseline use of medications used to treat osteoporosis (O.R. = 1.36, p = .0146). The total number of non-HRT medication classes used during the 45 days preceding the baseline survey was also significantly associated with discontinuation – women who used higher numbers of medications were less likely to discontinue HRT (O.R. for each additional therapeutic class = 0.95, p = .0021).

**Table 2:** Determinants of HRT discontinuation: results of propensity analysis

			Multivariate Logistic Regression Results*				
			
Variable	HRT Continuers (N = 1342)	HRT Discontinuers (N = 1015)	Parameter Estimate (β)	Std. Error of β	Adjusted Odds Ratio	95% CI for Odds Ratio	*P*-value
Age							
Age in years (mean)	75.5	75.5	0.0081	0.0075	1.041	0.993–1.023	.2847

Race							
White (%) †	96.7	95.6	--	--	1.000	--	--
Black (%)	2.7	3.5	0.3536	0.2527	1.424	0.868–2.337	.1616
Other race (%)	0.6	1.0	0.2903	0.4992	1.337	0.503–3.556	.5609

Income							
Annual income in thousands (mean)	$13.805	$13.999	0.0537	0.0245	1.055	1.006–1.117	.0283

Marital status							
Widowed, divorced, or never married (%) †	80.6	80.5	--	--	1.000	--	--
Currently married (%)	19.4	19.5	-0.1327	0.1384	0.876	0.668–1.149	.3378

Education							
12 or more years of education (%)	60.5	63.5	0.0937	0.0899	1.098	0.921–1.310	.2971

Residence type							
Nursing or personal care facility (%)	1.6	1.0	-0.4390	0.4067	0.645	0.291–1.431	.2804

Urban/rural residence							
Semi-rural or rural residence (%)	33.6	35.5	0.1526	0.0913	1.165	0.974–1.393	.0948

Alcohol use							
Current alcohol user (%)	26.5	24.4	-0.1335	0.1008	0.875	0.995–1.022	.1854

Smoking history							
Past or present smoker (%)	34.7	33.0	-0.0573	0.0924	0.944	0.788–1.132	.5352

Type of HRT used at baseline							
Unopposed estrogen (%) †	89.5	74.2	--	--	1.000	--	--
Combination estrogen/progestin (%)	10.5	25.8	1.1100	.1171	3.034	2.412–3.817	.0001

Baseline non-HRT prescription drug use							
Any cardiovascular drug use (%)	78.8	79.9	0.2265	0.1141	1.254	1.003–1.568	.0471
Any osteoporosis treatment (%)	12.2	15.4	0.3100	0.1270	1.363	1.063–1.749	.0146
Total number of non-HRT drug classes (mean)	6.3	5.9	-0.0507	0.0165	0.951	0.920–0.982	.0021

Baseline HRQOL							
Days that physical health was not good (mean)	6.3	6.3	0.0086	0.0069	1.009	0.995–1.022	.2174
Days that mental health was not good (mean)	3.3	3.0	-0.0042	0.0067	0.996	0.983–1.009	.5323
Healthy days (mean) ‡	21.9	22.3	--	--	--	--	--
Days that health interfered (mean)	4.2	3.8	-0.0113	0.0077	0.989	0.974–1.004	.1432
Days that pain made it hard (mean)	6.3	6.4	0.0060	0.0064	1.006	0.993–1.019	.3514

* All parameter estimates are adjusted for two-way interaction terms involving age, income, marital status, alcohol use, smoking history, prescription drug use, and baseline HRQOL.

† Reference group

‡ Because the healthy days variable is a composite of the physical and mental days measures, it was omitted from the logistic regression.

### Impact of discontinuation on HRQOL

As discussed above, HRT continuers and discontinuers were matched on the basis of the following factors: 1) the propensity scores obtained from the multivariate logistic regression, 2) the first principal component scores for the combined baseline HRQOL measures, 3) age group, and 4) type of HRT used at baseline. Of the 2,357 women present in the original sample, 1,770 respondents (75.1%) were successfully matched using these criteria. The final matched sample included 65.4% of the original sample of HRT continuers, and 86.5% of the original sample of discontinuers. Relaxing the matching algorithm requirements would have resulted in a higher number of matched subjects, but would have provided less control for selection bias.

**Figure 1:**
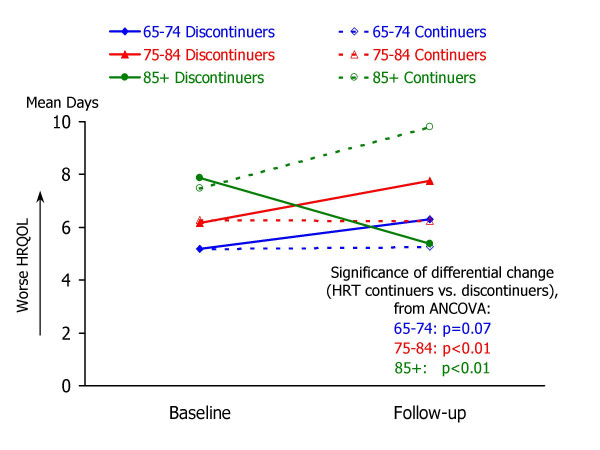
**Mean number of days that physical health was not good, by study occasion, age group, and HRT status. **Raw means by study occasion are presented for each age group (65–74, 75–84, and 85+) and HRT continuation group (continuers vs. non-continuers). For each age group, the statistical significance of the HRT continuation group difference in HRQOL change (from ANCOVA analyses; see Table 3) is also shown.

Table 3 summarizes the results of analysis of covariance to examine associations between HRT discontinuation and changes in HRQOL. For each of the follow-up HRQOL measures, mean values for HRT continuers and discontinuers are shown for three groups: 65–74 years, 75–84 years, and 85 years or older. Each ANCOVA model predicted follow-up HRQOL score from the corresponding baseline HRQOL measure and HRT group. Ancillary information to accompany the ANCOVA results in Table 3 is shown in Figures 1 through 5, which present the adjusted mean change in HRQOL for each measure by age group, while controlling for baseline HRQOL scores.

**Table 3:** Results of ANCOVA to compare HRQOL change in HRT continuers and discontinuers, by age group

			Raw Mean Scores								
								
			Continuers		Discontinuers						
								
Follow-up Measure	Age Group	N	Time 1	Time 2	Time 1	Time 2	Source of Variation	Sum of Squares	DF	F Value	Significance of F
*Number of days that physical health was not good*	65–74	810	5.19	5.27	5.17	6.31	Baseline physical days	19431.83	1	286.97	<.0001
							HRT group	218.26	1	3.22	.0730
	75–84	820	6.27	6.24	6.18	7.77	Baseline physical days	25852.59	1	348.90	<.0001
							HRT group	510.31	1	6.89	.0088
	85+	140	7.49	9.81	7.86	5.39	Baseline physical days	4130.90	1	50.34	<.0001
							HRT group	759.71	1	9.26	.0028

*Number of days that mental health was not good*	65–74	810	2.53	2.67	2.75	3.78	Baseline mental days	12963.01	1	309.92	<.0001
							HRT group	190.93	1	4.56	.0329
	75–84	820	2.49	3.67	2.52	3.12	Baseline mental days	11864.39	1	256.49	<.0001
							HRT group	67.51	1	1.46	.2274
	85+	140	2.93	5.15	2.17	1.99	Baseline mental days	1402.63	1	23.85	<.0001
							HRT group	287.32	1	4.88	.0287

*"Healthy days" composite*	65–74	810	23.43	23.30	23.43	21.97	Baseline healthy days	28043.55	1	340.82	<.0001
							HRT group	348.18	1	4.23	.0400
	75–84	820	22.41	21.81	22.59	20.89	Baseline healthy days	34431.84	1	388.18	<.0001
							HRT group	222.31	1	2.51	.1138
	85+	140	20.81	17.96	20.44	22.82	Baseline healthy days	5494.08	1	53.96	<.0001
							HRT group	914.52	1	8.98	.0032

*Number of days that physical or mental health interfered with activities*	65–74	810	3.22	3.40	3.17	4.89	Baseline interference days	14886.08	1	256.99	<.0001
							HRT group	455.88	1	7.87	.0051
	75–84	820	3.63	4.70	3.77	5.20	Baseline interference days	19210.32	1	303.58	<.0001
							HRT group	35.08	1	0.55	.4568
	85+	140	5.15	7.46	4.36	5.14	Baseline interference days	4440.59	1	54.06	<.0001
							HRT group	121.65	1	1.48	.2257

*Number of days that pain interfered with activities*	65–74	810	5.71	5.29	5.67	6.91	Baseline pain days	29680.32	1	446.86	<.0001
							HRT group	539.67	1	8.13	.0045
	75–84	820	5.80	7.02	5.88	7.65	Baseline pain days	33113.98	1	425.40	<.0001
							HRT group	67.38	1	0.87	.3525
	85+	140	7.54	8.46	8.44	6.74	Baseline pain days	4637.67	1	53.90	<.0001
							HRT group	169.95	1	1.98	.1621

**Figure 2:**
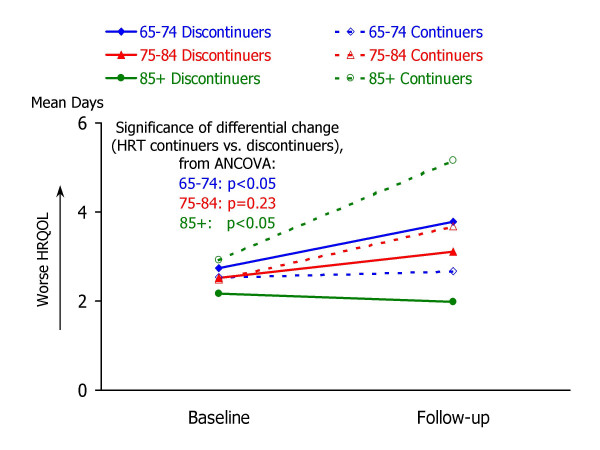
**Mean number of days that mental health was not good, by study occasion, age group, and HRT status. **Raw means by study occasion are presented for each age group (65–74, 75–84, and 85+) and HRT continuation group (continuers vs. non-continuers). For each age group, the statistical significance of the HRT continuation group difference in HRQOL change (from ANCOVA analyses; see Table 3) is also shown.

**Figure 3:**
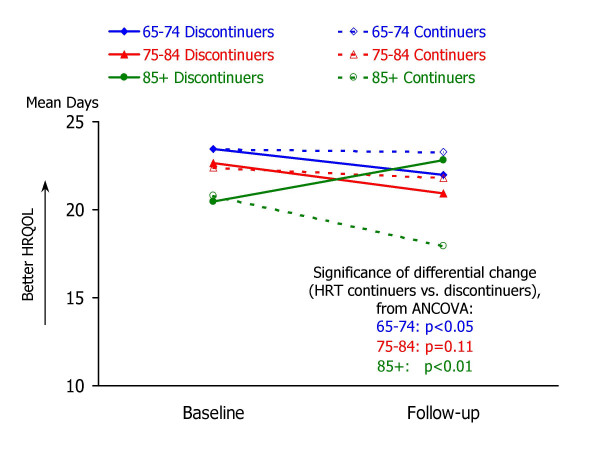
**Mean number of "healthy days", by study occasion, age group, and HRT status. **Raw means by study occasion are presented for each age group (65–74, 75–84, and 85+) and HRT continuation group (continuers vs. non-continuers). For each age group, the statistical significance of the HRT continuation group difference in HRQOL change (from ANCOVA analyses; see Table 3) is also shown.

**Figure 4:**
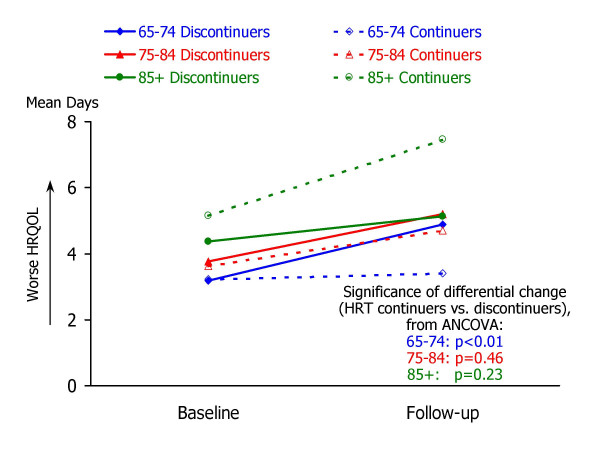
**Mean number of days that health interfered with activities, by study occasion, age group, and HRT status. **Raw means by study occasion are presented for each age group (65–74, 75–84, and 85+) and HRT continuation group (continuers vs. non-continuers). For each age group, the statistical significance of the HRT continuation group difference in HRQOL change (from ANCOVA analyses; see Table 3) is also shown.

**Figure 5:**
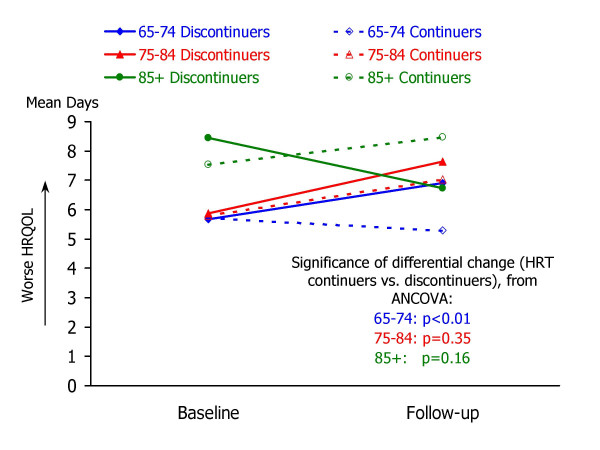
**Mean number of days that pain made activities difficult, by study occasion, age group, and HRT status. **Raw means by study occasion are presented for each age group (65–74, 75–84, and 85+) and HRT continuation group (continuers vs. non-continuers). For each age group, the statistical significance of the HRT continuation group difference in HRQOL change (from ANCOVA analyses; see Table 3) is also shown.

Striking age differences are apparent in the associations between HRT group and follow-up HRQOL, controlling for baseline HRQOL. Within the youngest group of women – aged 65–74 – HRT discontinuation was associated with a significant increase in the days in which mental health was not good (adjusted mean change = 1.1 days, p = .0329), fewer healthy days (adjusted mean change = -1.5 days, p = .0400), more days in which physical or mental health interfered with activities (adjusted mean change = 1.7 days, p = .0051), and more days in which pain made it hard to do routine activities (adjusted mean change = 1.2 days, p = .0045).

Among women aged 75–84, HRT discontinuers reported more days at follow-up in which their physical health was not good (adjusted mean change = 1.6 days, p = .0088). No other significant differences in HRQOL between HRT continuers and discontinuers were found in this age group.

In contrast to the apparent declines in HRQOL experienced by younger women who discontinued HRT, women aged 85 or older who discontinued HRT appeared to experience improvements in several HRQOL measures, while older continuers experienced declines. As shown in Table 2 and Figures 1 through 5, HRT discontinuers in this age group showed fewer days in which physical health was not good at follow-up, compared to baseline, while HRT continuers reported more "not good" physical days relative to baseline (adjusted mean changes for HRT continuers and discontinuers were 2.2 and -2.4, respectively; p = .0028). HRT continuers aged 85 or older also reported an increase in days that mental health was not good, while discontinuers experienced a slight decline (2.4 vs. -0.4, p = .0287). For the composite healthy days measure, HRT continuers experienced a mean decline of 2.8 days healthy days, while discontinuers exhibited a mean increase of 2.3 healthy days (p = .0032). In this oldest age group, HRT continuers and discontinuers did not differ significantly in the number of days at follow-up that health interfered with activities or that pain made it hard to do activities.

## Discussion

The goal of this study was to assess changes in health-related quality of life associated with discontinuation of HRT in a sample of elderly women. To our knowledge, no studies to date have specifically examined the impact of HRT discontinuation on quality of life. Given the large number of women who have either discontinued or will discontinue HRT, it is important to understand the associations between HRT cessation and HRQOL. The results of this study suggest that HRT discontinuation is associated with changes in HRQOL, but the direction and magnitude of these changes vary according to age. While HRT discontinuation was associated with apparent HRQOL declines among younger women in this study, women aged 85 or older appeared to experience improvements in HRQOL following HRT cessation.

The HRT discontinuation rate of 43% observed in this study is consistent with other recent reports. A recent study of U.S. national HRT prescribing rates found that overall HRT use declined by 38% between 2002 and 2003, with higher declines observed for combination therapy than for unopposed estrogen therapy [7]. The majority of HRT users in the present study used unopposed estrogen, warranted only for women without uteri. The high observed rate of unopposed estrogen use in this sample is supported by a 2000 PACE survey which found that, while 40% of all female respondents reported a prior hysterectomy, 75% of current HRT users reported having had a hysterectomy. These results suggest that hysterectomy history may play an important role in prescribers' decisions to initiate or continue HRT in elderly women.

Depending on the specific measure examined, HRT discontinuers aged 65–74 averaged an increase of one to two days per month in which HRQOL was suboptimal. The results described here do not explain what factors may have mediated this decline. Possibilities include acute vasomotor symptoms, such as flushing, which have been shown in prior studies to be important mediators of depression in menopausal women [31]. Alternatively, there may be more complex physiological effects of HRT discontinuation that affect mood, pain perception, or other aspects of perceived health.

The BRFSS HRQOL measures employed in this study have been previously shown to predict morbidity and mortality in the PACE population [32]. Based on the significant associations of these measures with HRT discontinuation it is plausible that HRT cessation may affect risk for some adverse outcomes, although data needed to address this question are not yet available. Recent work suggests that HRT discontinuation is associated with reduced bone density and increased fracture risk [33,34]. On the other hand, given the risks for breast cancer and cardiovascular disease that appear to be attributable to HRT, discontinuation may result in reduced risk for these outcomes. Clearly, more research is needed to explore the complex relationships among age, HRT cessation, HRQOL, and specific health outcomes.

A strength of this study is that it takes advantage of a natural experiment afforded by the availability of repeated survey data, which were collected during the general time period in which large numbers of women discontinued HRT. However, an important caveat regarding our findings is that our study design compared women who had either continued or discontinued HRT as of their follow-up survey date, but did not model the time course of HRQOL change following HRT discontinuation. Therefore, although the time interval between the baseline and follow-up surveys was approximately one year for all study subjects, the interval between HRT cessation and the follow-up survey response date could range from approximately one month to approximately one year. We conducted additional analyses to examine the impact of time since HRT cessation on HRQOL among discontinuers. Those analyses found no gradient in HRQOL change associated with the time elapsed since discontinuation. However, sample size considerations limited our statistical power to detect such differences. Further research is needed to model the time course of HRT discontinuation and its impact on HRQOL, and to examine whether short-term changes in HRQOL following cessation persist over time.

Due to the greater age and burden of illness present in the PACE population, the results of this study are limited in their generalizability to other populations, including younger women. These unique features of PACE, however, provide a valuable opportunity to examine the impact of HRT use and HRT discontinuation on elderly women. Most studies of HRT use have focused on women in the immediate postmenopausal years rather than elderly women. The present study, therefore, provides new information about the impact of HRT use and cessation on elderly women. The mean age of HRT users in this study was 75.5 years, and HRT users' ages at baseline ranged from 65 to 102. Although the large number of very old HRT users may be somewhat surprising, it is consistent with unpublished research indicating that 28% of PACE HRT users in 2000 were aged 80 or older. The same study found that, among current HRT users, the mean self-reported duration of use was 17.8 years, and 25% of current users reported that they had used HRT for 28 years or longer. These results highlight the need for awareness regarding the prevalence of very long-term HRT use by elderly women for whom menopause may have occurred several decades in the past.

A number of statistical limitations that could have a bearing on the results reported here should be noted. In this observational study, we sought to reduce selection bias through the use of propensity scores and by matching continuers with discontinuers on the basis of multiple factors. Comparison of the baseline HRQOL means for continuers and discontinuers suggested that the groups were well balanced in terms of baseline HRQOL. One limitation of this study, however, is that despite the statistical procedures employed to reduce bias, it is still possible that women who discontinued HRT differed in unmeasured ways from women who continued HRT. Conversely, because we matched on multiple variables as well as the propensity score, another statistical limitation relates to possible overmatching, as discussed by Rothman and Greenland [35]. To the extent that matching may have been performed on variables related to HRT use but not to HRQOL, statistical efficiency may have been reduced. Of greater concern is the possibility that some matching variables may have been directly related to HRQOL change, which could lead to additional bias [35].

Another limitation of this study is one that is inherent in any study relying on pharmacy claims data – HRT usage was inferred from prescription claim records. It is not known to what extent women who filled HRT prescriptions actually took the medication, or if the data recorded by the pharmacy at the point of sale accurately described patients' dosing instructions from their physicians. It is also possible that some women who did not fill prescriptions for HRT had access to the medication through other sources, such as samples received from physicians. Another important consideration relates to potential age-related measurement errors, such as recall bias, which may have affected women's perceptions of their HRQOL over the last 30 days. Age differences in women's expectations regarding the anticipated effects of HRT discontinuation could also be a factor. These limitations underscore the need for further studies to explicate the determinants and outcomes of HRT discontinuation.

Despite these limitations, the pattern of results observed in this study is an important reminder that even populations defined on the basis of age – such as PACE, for which the minimum eligible age is 65 – may include a broad range of ages with associated heterogeneity. Our results suggest that the response to HRT discontinuation among women aged 85 or older may be quite different from that of women in their 60's or 70's. The etiology of the age differences seen in this study is not known, and may reflect cohort effects, including the age-related measurement issues discussed above, or alternatively, the results may reflect physiological differences related to aging. Other recent work suggests that there are important age differences in the effects of estrogen on various physiological systems. For example, Brownley and her colleagues have recently reported differential associations between HRT and blood pressure according to the time elapsed since menopause [36]. There is also growing evidence from animal studies that the effects of estrogen replacement on neurological function may be attenuated with increasing age [37]. As discussed by Savonenko and Markowska [37], such findings suggest that aging processes may modulate the mechanisms by which estrogen exerts physiological effects.

Regardless of the mechanisms that may explain the pattern of HRQOL changes reported in this study, the declines observed among younger HRT discontinuers emphasize the need for communication between clinicians and patients throughout the discontinuation process. Based on current evidence obtained from clinical trials, HRT increases risk for breast cancer, stroke, and other adverse health outcomes. On that basis, HRT discontinuation is rational and may provide important health benefits. Nevertheless, short-term changes in HRQOL may occur following HRT cessation, and strategies to optimize the discontinuation process are needed. For example, current recommendations for HRT discontinuation advocate a gradual cessation in which dosing is tapered over a three to six month period [38,39]. Ideally, future research efforts will evaluate differences in HRQOL change according to the intensity and duration of the cessation process.

## Conclusions

This is the first study to present information about the impact of HRT discontinuation on HRQOL in elderly women. The results of this study suggest that there are significant age differences in response to HRT discontinuation. We believe that the present study furthers knowledge regarding older women's health in several important ways. First, it provides new information about potential declines in quality of life that women in their 60's and 70's may experience shortly after discontinuation of HRT. Awareness of these potential changes may help clinicians and patients to prepare for the discontinuation experience. These results have implications for clinical practice, suggesting that more clinical and social support may be needed for older women discontinuing HRT. These findings also highlight the importance of communication between health care providers and patients throughout the discontinuation process. Secondly, the results of this study suggest that many women aged 85 or older, including long-term users of HRT, may be able to discontinue HRT with little or no negative impact on quality of life. In fact, some women in this age group may experience improvements in quality of life upon HRT cessation, although the mechanisms involved in the observed improvements are not yet understood.

Finally, this study also has implications for health policy in that it illustrates the value of health monitoring and surveillance as a component of health program administration. Health-related quality of life is an important factor that should be considered when making clinical decisions and developing health policies. By adding an optional survey to the application process, the PACE Program has demonstrated a commitment to understanding the health status and needs of its older patient population. The use of survey measures such as those described in this study provide a useful framework for the evaluation of clinical program initiatives, and also provide a valuable resource for addressing relevant research questions. We hope that the work described here may encourage administrators of other programs to consider adopting similar health surveillance strategies.

## Competing interests

The author(s) declare that they have no competing interests.

## Authors' contributions

DAH conceived of the study, carried out the analyses, and drafted the manuscript. CHG assisted in study conception and design, and helped to rewrite the manuscript. FMA contributed to the study design and conception, provided statistical advice, and helped to draft the manuscript. KEP assisted with survey data processing and reviewed the manuscript. TVB assisted in the design of the survey and reviewed the manuscript. MRG assisted with the study conception and provided pharmacological expertise throughout the study. All authors read and approved the final manuscript.

## Pre-publication history

The pre-publication history for this paper can be accessed here:


